# On Aphasia: Being a Contribution to the Subject of the Dissolution of Speech from Cerebral Disease

**Published:** 1888-12

**Authors:** 


*On Aphasia: being a Contribution to the Subject of the Dissolution of Speech from Cerebral Disease.* By JAMES ROSS, M.D., LL.D. Aber. London: J. & A. CHURCHILL. 1887.

If, as is indeed the case, apology is due to Dr. Ross for the delay which has occurred in reviewing his book, that delay has arisen from the fact that his book, though not large, is so admirable, and withal somewhat abstruse, as to have caused its reperusal to be deferred from month to month until a more convenient season. There are still points in connection with aphasia which, not having received their certain explanation, remain subjects for the exercise of individual opinion: if Dr. Ross does not hesitate to put forth his own views in a clear and philosophical manner, he also quotes, with foot-note references, the views of other neurologists also upon such topics as are still in question, as well as upon those whose solution is now placed beyond doubt. Against Dr. Broadbent's peculiar subdivision of the faculties of the mind concerned with speech, he delivers himself of a mild Philippic, and certainly has the best of the argument. There are a few excellent woodcuts and diagrams (how much deficiency of fact a clear diagram may hide, as someone has remarked), which serve thoroughly well their explanatory purpose. By no means the last word has been spoken upon the subject of aphasia; and in another edition, which we shall hope to see, incorporating the latest facts and views, Dr. Ross will be able to bring his work up to date, and correct the typographical errors which detract from the excellence of this valuable book.

